# Rapid and sensitive identification of RNA from the emerging pathogen, coxsackievirus A6

**DOI:** 10.1186/1743-422X-9-298

**Published:** 2012-11-30

**Authors:** Lei Zhang, Xinying Wang, Yanjun Zhang, Liming Gong, Haiyan Mao, Cen Feng, David M Ojcius, Jie Yan

**Affiliations:** 1 Zhejiang Provincial Center for Disease Control and Prevention, 630 Xincheng Road, Hangzhou, Zhejiang, 310051, P.R. China; 2 Division of Basic Medical Microbiology, State Key Laboratory for Diagnosis and Treatment of Infectious Diseases, the First Affiliated Hospital, Zhejiang University School of Medicine, Hangzhou, Zhejiang, 310003, P.R. China; 3 Department of Medical Microbiology and Parasitology, Zhejiang University School of Medicine, 866 Yu-Hang-Tang Road, Hangzhou, Zhejiang, 310058, P.R. China; 4 Health Sciences Research Institute and School of Natural Sciences, University of California, Merced, CA, 95343, USA

**Keywords:** Hand, Foot and mouth disease, Enterovirus, Coxsackievirus A6, Quantitative real-time RT-PCR

## Abstract

**Background:**

Hand, foot and mouth disease (HFMD) is caused by members of the family *Picornaviridae* in the genus *Enterovirus*. It has been reported that coxsackievirus A6 (CVA6) infections are emerging as a new and major cause of epidemic HFMD. Sporadic HFMD cases positive for CVA6 were detected in the mainland of China in recent years. To strengthen the surveillance of CVA6 infections and outbreak control, the clinical diagnosis is urgently needed to distinguish the CVA6 infection disease from other infections.

**Methods:**

In order to develop a sensitive quantitative real-time RT-PCR assay for rapid detection of CVA6 RNA, primers and probe were designed to target the VP1 gene segment of CVA6. The conservation of the target segment was firstly analyzed by bioinformatic technology. The specificity of the real-time RT-PCR was further confirmed by detecting other related viruses and standard curves were established for the sensitivity evaluation. The pharyngeal swab samples from the EV71 and CVA16 unrelated HFMD patients were applied for CVA6 detection through the established method.

**Results:**

Based on the primer–probe set to detect the target VP1 gene segment of CVA6, the quantitative real-time RT-PCR assay could discriminate CVA6 infection from other resemble viral diseases with a potential detection limit of 10 viral copies/ml. The specificity of the assay was determined by sequence alignment and experimentally tested on various related viruses. The standard curve showed that the amplification efficiency of templates with different concentrations of templates was almost the same (R^2^ >0.99). Evaluation of the established method with pharyngeal swabs samples showed good accordance with the results from serology diagnosis.

**Conclusion:**

This study is the first report developing a VP1 gene-based quantitative real-time RT-PCR for rapid, stable and specific detection of CVA6 virus. The real-time RT-PCR established in this study can be used as a reliable method for early diagnosis of CVA6 infection.

## Introduction

Hand, foot, and mouth disease (HFMD) is characterized by fever and vesicular eruptions on the hands, feet and mouth. HFMD is caused by members of the family *Picornaviridae* in the genus *Enterovirus*[1]. In recent years, the prevalence of HFMD in the Asia-Pacific region, especially in Southeast Asia, has greatly increased. In China, a rapid expansion of HFMD out-breaks has occurred since 2004 [2]. In 2011, the Chinese Center for Disease Control and Prevention (China CDC) confirmed 1,619,706 cases in Mainland China including 509 deaths. Most outbreaks of HFMD were associated with the coxsackievirus A16 (CVA16) and human enterovirus 71 (EV71); however, sporadic cases involving other members of the enterovirus A species have also been reported [3]. During Fall 2008, an outbreak of HFMD with onychomadesis (nail shedding) as a common feature occurred in Finland. And the CVA6 was confirmed as the causative agent [4]. In 2010, an outbreak of CVA6 associated HFMD occurred in Taiwan and some patients also presented with onychomadesis and desquamation following HFMD [5]. It was suggested that CVA6 infections may be emerging as a new and major cause of epidemic HFMD.

To strengthen the surveillance of CVA6 infections and outbreak control, an effective detection method for the CVA6 is required. According to the previous studies, the traditional method requires reference anti-sera and relies on virus culture. Neutralization tests are still considered to be the “gold standard” for the identification of the CVA6 [6,7]. Although an indirect immunofluorescence assay (IFA) improved upon the traditional method and helped hospital laboratories deal simply with many clinical specimens, sero-diagnosis produced cross-immune responses with other enteroviruses. The molecular methods that are based on PCR and sequencing are faster and more accurate [8,9]. Recently, reverse transcription-PCR (RT-PCR) and real-time PCR assays have been used for enterovirus detection [10,11]. But there is still no advanced molecular detection and identification method for the CVA6 infection.

The purpose of this research is to develop a real-time PCR method based on the VP1 gene for the detection of CVA6 virus. The ability to distinguish it accurately from other members of the *Enterovirus* genus would be valuable. Based on the unique target, real-time RT-PCR could provide a simple and convenient identification method for the CVA6 infection.

## Materials and methods

### Clinical samples and viruses

This study was approved by the ethics committee of Zhejiang provincial Center for Disease Control and Prevention (CDC), China. With the permission of their parents, a total of 20 pharyngeal swabs were collected from the children patients with EV71 and CVA16 unrelated HFMD. The samples were stored in 3–5 ml of preservation solution (Hanks solution containing 100 U/ml penicillin and 100 μg/ml streptomycin) at −70°C for analysis. Poliovirus, coxsackievirus, human enterovirus 71, echovirus, rotavirus, sapovirus, norovirus, astrovirus and enteral adenovirus strains used in this study were all provided by Zhejiang provincial CDC.

### Sequence analysis and designation of primers

VP1 gene sequences from CVA6 virus strains were downloaded from GenBank. The Primer Express soft-ware package (Applied Biosystems, CA, USA) was used for primer and probe designation. All the primers used in this study were synthesized by Invitrogen Co. (Invitrogen, Shanghai, China). The target segment from VP1 gene which used for CVA6 virus identification was further used for conservation analysis through BLAT (http://blast.ncbi.nlm.nih.gov/Blast.cgi) and Mega 4.0 software (Biodesign Institute, AZ, USA).

### T-A cloning and sequencing

Genomic RNA of CVA6 virus was extracted by using the Virus Nucleic Acid Extraction Kit (Roche, Basel, Switzerland) and then eluted in TE buffer. A High Fidelity RT-PCR Kit (TaKaRa, Dalian, China) was used to amplify the target gene segment. According to the provided standard manipulation, the total volume per PCR was 50 μl which included 10 pmol of each of the primers CAF and CAR (Table 1), 25 μl 2× One Step RT-PCR Buffer III, 1 μl TaKaRa Ex TagHS (5 U/μl), 1 μl PrimeScript RT Enzyme Mix II, 4 μl total RNA and 17 μl RNase Free dH_2_O. The reverse transcription reaction was performed by incubation at 42°C for 5 min and terminated by incubation at 95°C for 10 s, then followed by 40 cycles of PCR amplification at 95°C for 5 s, 60°C for 34 s. The products were detected in 1.5% Ethidium Bromide pre-stained agarose gel after electrophoresis. In order to obtain the accurate sequence data, the PCR product was purified and cloned into the plasmid pMD19-T by using the T-A clone kit (TaKaRa). The recombinant plasmids (pMD19-T-VP1) were used for sequencing by Invitrogen Co. DNA sequences were compared to the National Center for Biotechnology Information (NCBI) database.

**Table 1:** Nucleotide sequences of the primers and probe used in this study

**Primer/probe**	**Sequence (5**^**′**^**to 3**^**′**^**)**	**genomic position**	**PCR product size (bp)**
CAF	CAAGCTGCAGAAACGGGAG	2572- 2590	103
CAR	GCTCCACACTCGCCTCATT	2656- 2674	
CAP	FAM-ACCCCGTTTCGATTCATCACACA-BHQ1	2632-2654	

### TaqMan Real time RT-PCR reaction

TagMan Real time RT-PCR reaction was performed by using the One Step PrimeScript RT-PCR Kit (TaKaRa, Dalian, China). Each reaction mixture consisted of 10 pmol of the primers CAF and CAR, 20 pmol of the probe CAP (Table 1), and 4 μl of template RNA in a final volume of 50 μl. According to the instructions, RT-PCR reactions were performed with the same condition as the conventional RT-PCR, which was described above. Fluorescence signals were measured every cycle at the end of the annealing step.

### Sensitivity and specificity of real time RT-PCR assays

Instead of using CVA6 genomic nucleic acid, plasmid pMD19-T-VP1 was applied for the real-time RT-PCR sensitivity analysis here. DNA templates from tenfold serial dilutions of pMD19-T-VP1 were analyzed by the real-time RT-PCR, and the results were used to generate a standard curve.

In order to evaluate the specificity of the real time RT-PCR established in this study, genomic nucleic acid extracts from the poliovirus typeI-III; coxsackievirus A16, B1, B5, B3; human enterovirus 71; echovirus 6, 30; rotavirus A; sapovirus; norovirusII; astrovirus and enteral adenovirus strains were prepared and used as templates. The positive recombinant plasmid generated previously, (pMD19-T-VP1) was used as positive control. These extracts were analyzed in duplicate reactions on an ABI 7500 sequence detection system by the established amplifying standards. A CT value of 40 (maximum number of PCR cycles run) indicated that genomic nucleic acid from the test strains were not detected.

### Real-time RT-PCR detection of clinical samples

Genomic nucleic acid extracted from all the pharyngeal swabs samples were used as templates. With the primers (CAF and CAR) and the TaqMan probe, the real-time RT-PCR developed in this study was applied for CVA6 identification. The amplification procedure was the same as described above.

## Results

### Sequence analysis

**Figure 1:**
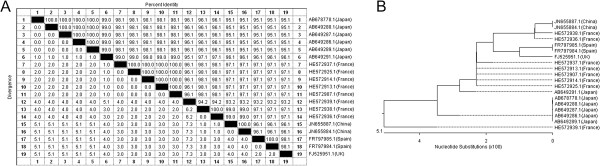
**Homology comparison of the target VP1 gene segment sequences from different CVA 6 virus strains. A**: The similarities of the target VP1 gene segment sequences from the CVA6 virus strains of China, Japan, France, Spain and UK were 93.2-100%. **B**: Phylogenetic analysis result showed the average variability among these sequences was 5.1%.

After acquiring the VP1 gene from the GeneBank, the sequence of the VP1 gene from Human CVA6 virus strain Shizuoka 18 (GenBank NO.: AB678778.1) was subjected to primer design by using Primer Express software. The primer and probe sequences were generated and the target segment was then applied for homology analysis through blast in NCBI. We found that the sequence of the target gene segment was only conserved present in Human CVA6 virus strains. The similarities of the target VP1 gene segment sequences from the CVA6 strains of China (GenBank NO.: JN655887.1, JN655884.1), Japan (GenBank NO.: AB678778.1, AB649288.1, AB649287.1, AB649286.1, AB649289.1, AB649291.1), France (GenBank NO.: HE572937.1, HE572925.1, HE572914.1, HE572913.1, HE572907.1, HE572939.1, HE572938.1, HE572936.1), Spain (GenBank NO.: FR797985.1, FR797984.1), UK(GenBank NO.: FJ525951.1) was 93.2-100% (Figure 1A) and the average variability was 5.1% as calculated by MegAlign procedure (Figure 1B). The conservation of the target VP1 gene segment sequence of the Human CVA6 virus suggests that it can be a useful marker for the Human CVA6 virus detection and identification.

### PCR amplification and TA clone

**Figure 2:**
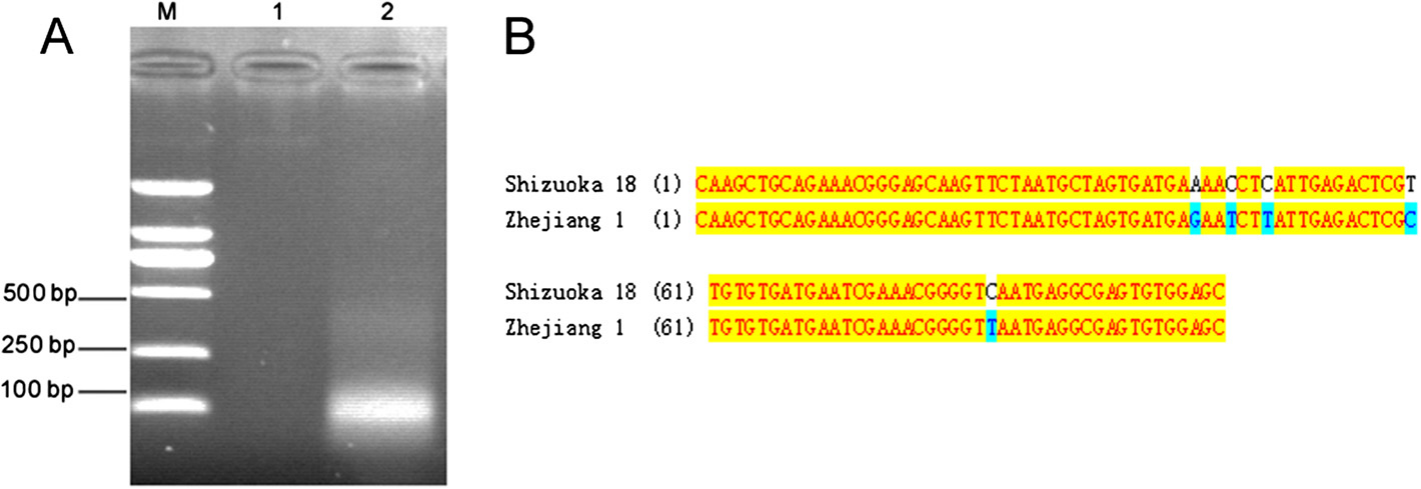
**PCR amplification and sequencing. A**: M: Marker; 1: Negative control; 2: PCR amplification product of the target VP1 gene segment from CVA 6 virus using primers CAF and CAR (103 bp). **B**: Comparison of the amplified sequence of the target VP1 gene segment with the reference sequence from strain Shizuoka 18 (GenBank NO.: AB678778.1). The similarity between these sequences was 95.1%.

After PCR amplification using genomic nucleic acid from Human CVA6 as template, product was visualized on a 1.5% agarose gel (Figure 2A). According to the size of the target VP1 gene segment described above, the size of the amplification product was correct (103 bp). The results also suggested the specificity of the primers. The PCR product was purified and cloned into the plasmid pMD19-T. The generated recombinant plasmid pMD19-T-VP1 was further confirmed by sequencing. After comparison with the reference sequence from human CVA6 strain Shizuoka 18, we observed that the similarity between them was 95.1% (Figure 2B). Several site mutations have been found in the sequence of the target VP1 gene segment, However, it showed little impact on the primers conjugation. It also indicated that the sequence of the target VP1 gene segment from the human CVA6 of different areas showed small variability as described above.

### Sensitivity and specificity of real time RT-PCR assay

**Figure 3:**
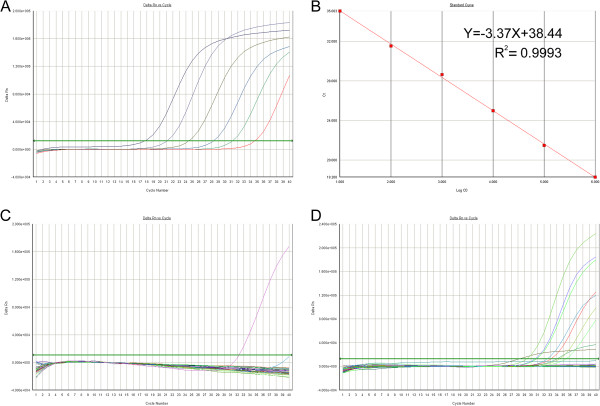
**Sensitivity, specificity analysis of the real-time RT-PCR and clinical samples detection. A**: Real-time RT-PCR amplification plot of the CVA6 viral gene segment. A PCR baseline subtractive curve fit view of the data is shown with relative fluorescence units (RFU) plotted against cycle number. The default setting of 10 times the standard deviation of fluorescence in all wells over the baseline cycles was used to calculate the threshold cycle or Ct value for a positive reaction (horizontal line). **B**: Standard curve analysis of the RNA amplification plots with Ct values plotted against starting copy numbers. Typical amplification plot derived from serial tenfold dilutions of recombinant plasmid pMD19-T-VP1 ranged from 10^6 ^to 10^1 ^copies/ml (R^2^ = 0.9993). **C**: Specificity analysis of the Real time RT-PCR was performed by using the genomic nucleic acid from poliovirus typeI-III; coxsackievirus A16, B1, B5, B3; human enterovirus 71; echovirus 6, 30; rotavirus A; sapovirus; norovirusII; astrovirus and enteral adenovirus strains as control. For the primer–probe sets, there were no positive results obtained except the RNA transcripts of the CVA6 virus. **D**: Clinical samples were identified by the real-time RT-PCR. By using the recombinant plasmid pMD19-T-VP1 as control, RNA transcripts from eight clinical samples give a typical amplification plot.

Based on the TagMan probe, a Real-time PCR method has been established for the detection of Human CVA6 virus in pharyngeal swabs samples. In order to estimate the sensitivity of the Real Time RT-PCR, series dilutions of recombinant plasmid (pMD-19-T-VP1) DNA templates generated previously were used for standard curve analysis, each in triplicate. The detection range of the real-time RT-PCR method was at least from 10^1^ to 10^6^ copies/ml (Figure 3A). And slight variations were seen in the amplification efficiency among different templates, which suggested that the rea-time RT-PCR was stable in detection of the target VP1 gene segment. Data were then subjected to log-linear analysis to generate a standard curve for calculation of unknowns (Figure 3B). The standard curves regularly exhibited high R^2^ values (>0.99).

First, GeneBank was explored to find out whether the primer sequences paired with sequences other than the target VP1 gene segment from human CVA6, but after searching through BLAST, no close hits from gene segment other than the target VP1 gene segments from human CVA6 virus were found. Then, the specificity of the established real-time RT-PCR detection system was tested by using the genomic nucleic acid from other virus of the same genus or virus which may cause the resemble symptoms. Poliovirus typeI-III; coxsackievirus A16, B1, B5, B3; human enterovirus 71; echovirus 6, 30; rotavirus A; sapovirus; norovirusII; astrovirus and enteral adenovirus have been used for specificity evaluation here, and we found that only the human CVA6 virus gave CT values over the detection limit after real-time RT-PCR detection (Figure 3C).

### Identification of bacterial strains isolated from clinical samples

**Figure 4:**
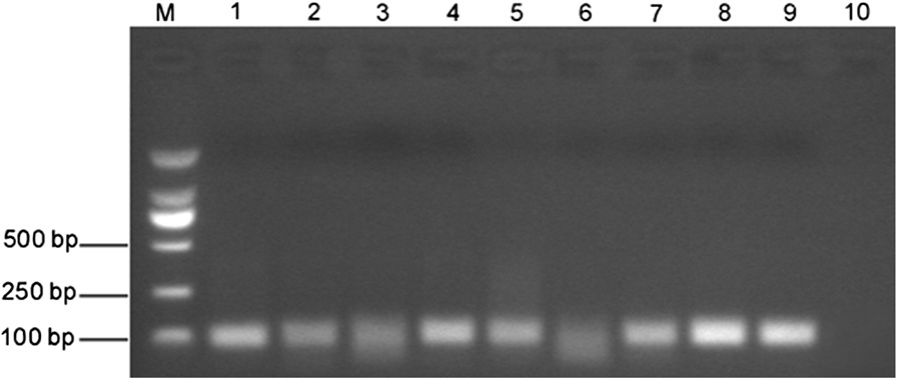
**Confirmation the identification results of clinical samples. **M: Marker; 1–8: The PCR products of the positive clinical samples; 9–10: Positive control and negative control. The PCR products were visualized on an agarose gel and confirmed to be in the correct size (103 bp).

Pharyngeal swabs samples were obtained from the suspected HFMD patients. Twenty samples which were first discriminated from the human EV71 and CVA16 infections were further identified by the real-time RT-PCR developed in this study. By using the recombinant plasmid pMD-19-T-VP1 DNA as positive control, real-time RT-PCR identification results showed that eight samples gave a positive amplification (Figure 3D). The PCR products were also visualized on an agarose gel to confirm that they were the correct size and sequenced to confirm their identity (Figure 4). The serum samples of these patients were collected during the convalescent period and applied for the IFA tests. The serology diagnosis showed the same results as detection by the real-time RT-PCR (data not shown). This suggested that the real-time RT-PCR not only takes less time at lower cost but also provides a precise approach for the virus identification.

## Discussion

Human enteroviruses have more than 90 serotypes, which cause a wide spectrum of acute febrile diseases among infants and children. Hand, foot, and mouth disease; aseptic meningitis; respiratory tract illnesses; herpangina and otitis media are all could be caused by the infection of the human enteroviruses [12]. HFMD has become an important issue for China now [13]. A rapid expansion of HFMD outbreaks has occurred since 2004. People are still mainly concerned with EV71 and CVA16, however, little is known about the other enterovirus infections and distributions [14].

It has been reported that *Enterovirus* CVA6 was a primary pathogen associated with HFMD during a nationwide outbreak in Finland in autumn of 2008 [15]. In 2010, an outbreak of CVA6 associated HFMD occurred in Taiwan and some patients presented with onychomadesis and desquamation following HFMD. HFMD cases were found positive for CVA6 was also detected in the mainland of China in recent years. To strengthen the CVA6 infections surveillance and outbreak control, useful diagnostic technologies are required in the clinical laboratories. The molecular methods that depend on PCR and sequencing are fast and accurate for CVA6 identification, but few associated research has been found previously.

Real-time RT-PCR is generally accepted as an accurate and cost-effective method for virus identification. We set out to design a quantitative real-time RT-PCR assay to identify and quantify CVA6 virus present in the clinical samples. VP1 gene has been widely used as the target for enteroviruses identification, which was also used here [16]. The Primer Express software has been applied for primer designation. After bioinformatics analysis, we found the target segment only conserved present in the CVA6 strains. The similarity among the sequences from different strains is above 90%. So it is considered to be a good target for CVA6 virus identification.

The real-time RT-PCR assay for diagnosis of CVA6 virus infection has many advantages over conventional methods, either for detection limit or specificity. The standard curve showed that the amplification efficiency of templates with different concentrations of DNA was almost the same and the maximum sensitivity was 10 copies/ml at least. Specificity investigation of the real-time qRT-PCR assay revealed that only the templates of the CVA6 RNA showed a positive detection results.

Finally, real-time qRT-PCR established in this study was further applied to identify the HFMD clinical samples. Interestingly, 40% of the EV71 and CVA16 unrelated HFMD clinical samples were identified as CVA6 virus infection. So the CVA6 virus seems to be a primary pathogen associated with HFMD besides EV71 and CVA16. Though the majority of HFMD cases are caused by EV71 and CVA16 infections here in China, when diagnosis of the HFMD cases shows RT-PCR positive for enterovirus but negative for EV71 and CVA16, it is necessary to use the method established here for further identification.

In conclusion, the real-time RT-PCR assay for CVA6 detection presented in this report provided a reliable tool for early clinical diagnosis, which will enhance the diagnostic capacity of epidemiological surveillance and guide clinical treatment for CVA6 infection.

## Competing interests

The authors declare that they have no competing interests.

## Authors' contributions

LZ, XW and DMO carried out the molecular genetic studies, participated in the sequence alignment and drafted the manuscript. LG and HM carried out the RT-PCR assays. CF participated in the sequence alignment. JY participated in the design of the study. YZ conceived of the study, and participated in its design and coordination. All authors read and approved the final manuscript.
